# Genomic evaluation for two-way crossbred performance in cattle

**DOI:** 10.1186/s12711-023-00792-4

**Published:** 2023-03-17

**Authors:** Quanshun Mei, Huiming Liu, Shuhong Zhao, Tao Xiang, Ole F Christensen

**Affiliations:** 1 Key Laboratory of Agricultural Animal Genetics, Breeding and Reproduction of Ministry of Education & Key Laboratory of Swine Genetics and Breeding of Ministry of Agriculture, Huazhong Agricultural University, Wuhan, 430070 China; 2 Center for Quantitative Genetics and Genomics, Aarhus University, C. F. Møllers Allé 3, 8000 Aarhus C, Denmark; 3 SEGES Cattle, Agrofood Park 15, 8200 Aarhus N, Denmark

## Abstract

**Background:**

Dairy cattle production systems are mostly based on purebreds, but recently the use of crossbreeding has received increased interest. For genetic evaluations including crossbreds, several methods based on single-step genomic best linear unbiased prediction (ssGBLUP) have been proposed, including metafounder ssGBLUP (MF-ssGBLUP) and breed-specific ssGBLUP (BS-ssGBLUP). Ideally, models that account for breed effects should perform better than simple models, but knowledge on the performance of these methods is lacking for two-way crossbred cattle. In addition, the differences in the estimates of genetic parameters (such as the genetic variance component and heritability) between these methods have rarely been investigated. Therefore, the aims of this study were to (1) compare the estimates of genetic parameters for average daily gain (ADG) and feed conversion ratio (FCR) between these methods; and (2) evaluate the impact of these methods on the predictive ability for crossbred performance.

**Methods:**

Bivariate models using standard ssGBLUP, MF-ssGBLUP and BS-ssGBLUP for the genetic evaluation of ADG and FCR were investigated. To measure the predictive ability of these three methods, we estimated four estimators, bias, dispersion, population accuracy and ratio of population accuracies, using the linear regression (LR) method.

**Results:**

The results show that, for both ADG and FCR, the heritabilities were low with the three methods. For FCR, the differences in the estimated genetic parameters were small between the three methods, while for ADG, those estimated with BS-ssGBLUP deviated largely from those estimated with the other two methods. Bias and dispersion were similar across the three methods. Population accuracies for both ADG and FCR were always higher with MF-ssGBLUP than with ssGBLUP, while with BS-ssGBLUP the population accuracy was highest for FCR and lowest for ADG.

**Conclusions:**

Our results indicate that in the genetic evaluation for crossbred performance in a two-way crossbred cattle production system, the predictive ability of MF-ssGBLUP and BS-ssGBLUP is greater than that of ssGBLUP, when the estimated variance components are consistent across the three methods. Compared with BS-ssGBLUP, MF-ssGBLUP is more robust in its superiority over ssGBLUP.

**Supplementary Information:**

The online version contains supplementary material available at 10.1186/s12711-023-00792-4.

## Background

Crossbreeding is commonly used in many livestock production systems [1], especially for pig and poultry. For dairy and beef cattle, production systems are mostly based on purebreds, but recently the use of crossbreeding between dairy breed cows and beef breed bulls has received increased interest for a number of reasons [1]. In particular, meat production from crossbreds between dairy cows and beef bulls has a lower environmental footprint than that from beef cattle [2]. Furthermore, because the improved reproductive performance of dairy cows reduces the need for replacement heifers, some dairy cows in a herd can be inseminated with beef semen.

For livestock production systems that use crossbreds, although the breeding goal is to improve crossbred performance, selection usually takes place in the purebred lines [3], which is sub-optimal since the genetic performances of purebred (PB) and crossbred (CB) animals differ [4–6]. By reviewing the existing literature on the genetic correlations between the performances of purebred and crossbred pigs, Wientjes and Calus [7] found an average genetic correlation of 0.6, while based on the review of 14 studies on broilers and layers, Calus et al. [8] found an average genetic correlation of 0.71. These results indicate that it is meaningful to select for CB performance as well as for PB performance in crossbred production systems.

Since 2010, single-step genomic best linear unbiased prediction (ssGBLUP) has been used as a standard genomic selection (GS) method in the pig industry, and has shown a high predictive ability for both genotyped and non-genotyped animals [9–11]. However, when crossbred information is considered, ssGBLUP does not fit well due to the existence of genetic differences between breeds (i.e., allele frequency, linkage disequilibrium, and gametic phase) [12]. An alternative ssGBLUP, called breed-specific ssGBLUP (BS-ssGBLUP) that integrates purebred and crossbred information, was proposed by Christensen et al. [13] based on multiple breed-specific relationship matrices [14]. Xiang et al. [5] applied this method on real pig data and validated its superiority over ssGBLUP. However, other studies have not confirmed this superiority [15, 16]. Another method called metafounder ssGBLUP (MF-ssGBLUP) has been developed by Legarra et al. [17] to model genetic differences between breeds. The differences in the estimates of genetic parameters and predictive ability between these three methods have been investigated using pig data [5, 18] and simulated data [19], but not with data on crossbreds between dairy and beef cattle, thus more research is needed. In addition, to date, ssGBLUP and MF-ssGBLUP have been successfully used in crossbred cattle to estimate genetic parameters [20, 21], but not BS-ssGBLUP.

Thus, the objectives of this study were to (1) compare the estimates of genetic parameters in crossbred beef and dairy cattle for average daily gain (ADG) and feed conversion ratio (FCR) with ssGBLUP, MF-ssGBLUP and BS-ssGBLUP; and (2) evaluate the impact of these methods on the predictive ability for crossbred performance.

## Methods

### Data

All datasets were provided by SEGES Innovation Cattle and Nordic Cattle Genetic Evaluation. In this study, 4089 two-way crossbred calves (BH) with purebred Belgian Blue beef (BBL) sires and purebred Holstein dairy (HOL) dams were on test for about 1 month. During this period, feed intake was recorded for each animal, and body weight of each animal was recorded at both the start and end of the test period. ADG (kg/d) and FCR (kg/kg) of each animal within this period were calculated as the increase in body weight divided by number of days and the average daily feed intake divided by average daily gain, respectively. After data editing of feed intake and body weight records (see Additional file 1: Fig. S1), 2592 crossbred calves were retained, with ADG available for all the calves and FCR for 2306 calves. The birth dates of these calves ranged from June 1 2019 to December 1 2021. These 2592 crossbred animals originate from 67 sires and 2419 dams, with an average number of progeny per sire of 38.7 and per dam of 1.1, and the average size of paternal half-sibling families was 37.6. The average age of these calves was 207 days (standard deviation (SD) = 34 days) at the beginning of the test, and 243 days (SD = 33 days) at the end of the test. Descriptive statistics of the phenotypes are in Table 1.

**Table 1:** Descriptive statistics

Trait	Mean (SD)	Min	Max	Number of animals with phenotype	Number of animals with both phenotype and genotype	Number of animals in partial dataset (cut-off date, April 1 2021)	Number of animals in partial dataset (cut-off date, May 1 2021)
ADG (kg)	1.411 (0.345)	0.077	2.857	2592	1258	1944	2016
FCR	4.984 (1.751)	0.436	19.663	2306	1115	1786	1855

ADG: average daily gain; FCR: feed conversion ratio

Pedigree for the crossbred animals was traced back three generations, and included 30,643 animals with 846 BBL, 25,709 HOL and 4088 BH. Among these animals, 43 BBL and 882 HOL were genotyped with the EuroG 10K Bead chip, and 39 BBL, 1590 HOL, and 1780 BH were genotyped with the Eurogenomics 75K custom SNP chip. Among the parents of the BH, 52 BBL and 319 HOL were genotyped. For all genotyped animals, the procedure of filling-in missing genotypes and imputation from the EuroG 10K Bead chip to the Eurogenomics 75K custom SNP chip was done with the Beagle 5.2 software [22]. Quality control of the genomic data was done using the Plink software as follows [23]: first, we checked that no individuals had a call-rate lower than 90%; then, SNPs with a call-rate lower than 90%, SNPs with a minor allele frequency lower than 0.01, and SNPs that deviated strongly from the Hardy–Weinberg equilibrium within breed (p < 10–7) were removed. Finally, 4329 animals (81 BBL and 2468 HOL, and 1780 BH) and 48,777 SNPs were retained after quality control and imputation. The retained genotype data were phased with the Beagle 5.2 software [22].

### Statistical models

A bivariate animal model was used to estimate genetic parameters and breeding values for ADG and FCR. To construct the single-step relationship matrices, three methods, standard ssGBLUP, MF-ssGBLUP, and BS-ssGBLUP, were incorporated in the bivariate model, as follows.

### Standard ssGBLUP

With the aim of extending the marker-based relationship matrices to the non-genotyped animals, Legarra et al. [9] and Christensen and Lund [10] developed ssGBLUP.

The statistical bivariate model for ssGBLUP is:

1 $$\mathbf{y}=\mathbf{X}\mathbf{b}+\mathbf{Z}\mathbf{u}+\mathbf{e},$$

where ${\mathbf{y}}$ is the vector of phenotypic records for ADG and FCR in crossbred calves; $\mathbf{b}$ is the vector of fixed effects including the effects of sex, pen (during the experiment), herd-year-season (year and season of the testing period), and covariate of the weight at the start of the test for ADG and FCR; $\mathbf{u}$ is the vector of random additive genetic effects for ADG and FCR; ${\mathbf{e}}$ is the vector of the random residual error for ADG and FCR; ${\mathbf{X}}$ and ${\mathbf{Z}}$ are the corresponding incidence matrices.

It is assumed that the random effects follow normal distributions, i.e. $\mathbf{u}\sim \mathrm{N}(\boldsymbol{0}, {\sum }_{\mathrm{u}}\otimes \mathbf{H})$ and $\mathbf{e}\sim \mathrm{N}(\boldsymbol{0}, {\sum }_{\mathrm{e}}\otimes \mathbf{I})$, where $\mathbf{H}$ is the combined pedigree-based and marker-based relationship matrix presented below; $\mathbf{I}$ is the corresponding identity matrix; ${\sum }_{\mathbf{u}}$ is the genetic (co)variance matrix, ${\sum }_{\mathbf{e}}$ is the residual (co)variance matrix, and $\otimes$ denotes the Kronecker product. The (co)variance matrices are as follows:

$${\sum }_{\mathbf{u}}=\left[\begin{array}{cc}{\upsigma }_{{\mathrm{u}}_{\mathrm{ADG}}}^{2}& {\upsigma }_{{\mathrm{u}}_{\mathrm{ADG}}{\mathrm{u}}_{\mathrm{FCR}}}\\ \mathrm{sym}& {\upsigma }_{{\mathrm{u}}_{\mathrm{FCR}}}^{2}\end{array}\right],$$

$$\mathrm{and }\,{\sum }_{\mathbf{e}}=\left[\begin{array}{cc}{\upsigma }_{{\mathrm{e}}_{\mathrm{ADG}}}^{2}& {\upsigma }_{{\mathrm{e}}_{\mathrm{ADG}}{\mathrm{e}}_{\mathrm{FCR}}}\\ \mathrm{sym}& {\upsigma }_{{\mathrm{e}}_{\mathrm{FCR}}}^{2}\end{array}\right],$$

where ${\upsigma }_{{\mathrm{u}}_{\mathrm{ADG}}}^{2}$ is the additive genetic variance of ADG, ${\upsigma }_{{\mathrm{u}}_{\mathrm{FCR}}}^{2}$ is the additive genetic variance of FCR, ${\upsigma }_{{\mathrm{u}}_{\mathrm{ADG}}{\mathrm{u}}_{\mathrm{FCR}}}$ is the additive genetic covariance between ADG and FCR; ${\upsigma }_{{\mathrm{e}}_{\mathrm{ADG}}}^{2}$ is the residual variance of ADG, ${\upsigma }_{{\mathrm{e}}_{\mathrm{FCR}}}^{2}$ is the residual variance of FCR, and ${\upsigma }_{{\mathrm{e}}_{\mathrm{ADG}}{\mathrm{e}}_{\mathrm{FCR}}}$ is the residual covariance between ADG and FCR.

The combined pedigree-based and marker-based relationship matrix $\mathbf{H}$ is defined as [9, 10]:

$$\mathbf{H}=\left[\begin{array}{cc}{\mathbf{A}}_{\boldsymbol{11}}-{\mathbf{A}}_{\boldsymbol{12}}{\mathbf{A}}_{\boldsymbol{22}}^{-\boldsymbol{1}}{\mathbf{A}}_{\boldsymbol{21}}+{\mathbf{A}}_{\boldsymbol{12}}{\mathbf{A}}_{\boldsymbol{22}}^{-1}\mathbf{G}{\mathbf{A}}_{\boldsymbol{22}}^{-1}{\mathbf{A}}_{\boldsymbol{21}}& {\mathbf{A}}_{\boldsymbol{12}}{\mathbf{A}}_{\boldsymbol{22}}^{-\boldsymbol{1}}\mathbf{G}\\ \mathbf{G}{\mathbf{A}}_{\boldsymbol{22}}^{-1}{\mathbf{A}}_{\boldsymbol{21}}& (1-\upomega )\mathbf{G}+\upomega {\mathbf{A}}_{\boldsymbol{22}}\end{array}\right],$$

where $\mathbf{A}$ is the pedigree relationship matrix, $\mathbf{G}$ is the genomic realized relationship matrix; subscripts 1 and 2 stand for non-genotyped and genotyped animals, respectively; $\upomega$ is interpreted as the relative weight on the polygenic effect, which is set as 0.05 in this study as commonly done [24, 25].

The relationship matrix $\mathbf{G}$ was constructed as [26]:

$$\mathbf{G}=\frac{\mathbf{Z}\mathbf{Z}\mathbf{^{\prime}}}{\sum_{\mathrm{i}=1}^{\mathrm{m}}2{\mathrm{p}}_{\mathrm{i}}{\mathrm{q}}_{\mathrm{i}}},$$

where $\mathrm{m}$ is the number of SNPs, ${\mathrm{p}}_{\mathrm{i}}$ is the frequency of allele *A* at marker $\mathrm{i}$ and ${\mathrm{q}}_{\mathrm{i}}=1-{\mathrm{p}}_{\mathrm{i}}$; $\mathbf{Z}$ is the incidence matrix with elements of $2-2{\mathrm{p}}_{\mathrm{i}}$, $1-2{\mathrm{p}}_{\mathrm{i}}$ and $-2{\mathrm{p}}_{\mathrm{i}}$ for *AA*, *Aa*, and *aa*, respectively. Matrix $\mathbf{G}$ was adjusted to be compatible with matrix $\mathbf{A}$ as described by Christensen et al. [27].

### MF-ssGBLUP

To account for allele frequency in the base population and compatibility between the pedigree and genomic additive relationship matrices, Legarra et al. [17] developed a new method named MF-ssGBLUP, based on developments described in Christensen [27].

The statistical bivariate model for MF-ssGBLUP is:

2 $$\mathbf{y}=\mathbf{X}\mathbf{b}+\mathbf{Z}\mathbf{u}+\mathbf{e},$$

where ${\mathbf{y}}$, $\mathbf{b}$, $\mathbf{u}$, $\mathbf{e}$, $\mathbf{X}$ and $\mathbf{Z}$ are as defined above.

The difference between Eq. (1) and Eq. (2) is the definition of the additive genetic relationship matrix. For Eq. (2), it is assumed that the random effects follow normal distributions, i.e. $\mathbf{u}\sim \mathrm{N}(\boldsymbol{0}, {\sum }_{\mathrm{u}}\otimes {\mathbf{H}}_{\mathbf{M}\mathbf{F}})$, where ${\mathbf{H}}_{\mathbf{M}\mathbf{F}}$ is the combined pedigree-based and marker-based metafounder relationship matrix, and ${\sum }_{\mathbf{u}}$ contains the genetic variance and covariance parameters.

The matrix ${\mathbf{H}}_{\mathbf{M}\mathbf{F}}$ is defined as:

$${\mathbf{H}}_{\mathbf{M}\mathbf{F}}=\left[\begin{array}{cc}{\mathbf{A}}_{\boldsymbol{11}}^{{\varvec{\Gamma}}}-{\mathbf{A}}_{\boldsymbol{12}}^{{\varvec{\Gamma}}}{{\mathbf{A}}_{\boldsymbol{22}}^{{\varvec{\Gamma}}}}^{-1}{\mathbf{A}}_{\boldsymbol{21}}^{{\varvec{\Gamma}}}+{\mathbf{A}}_{\boldsymbol{12}}^{{\varvec{\Gamma}}}{{\mathbf{A}}_{\boldsymbol{22}}^{{\varvec{\Gamma}}}}^{-\boldsymbol{1}}{\mathbf{G}}^{0.5}{{\mathbf{A}}_{\boldsymbol{22}}^{{\varvec{\Gamma}}}}^{-\boldsymbol{1}}{\mathbf{A}}_{21}^{{\varvec{\Gamma}}}& {\mathbf{A}}_{12}^{{\varvec{\Gamma}}}{{\mathbf{A}}_{22}^{{\varvec{\Gamma}}}}^{-\boldsymbol{1}}{\mathbf{G}}^{0.5}\\ {\mathbf{G}}^{0.5}{{\mathbf{A}}_{\boldsymbol{22}}^{{\varvec{\Gamma}}}}^{-1}{\mathbf{A}}_{\boldsymbol{21}}^{{\varvec{\Gamma}}}& (1-\upomega ){\mathbf{G}}^{0.5}+\upomega {{\mathbf{A}}_{\boldsymbol{22}}^{{\varvec{\Gamma}}}}^{-\boldsymbol{1}}\end{array}\right],$$

where $\upomega$ is as defined above; ${\mathbf{A}}^{{\varvec{\Gamma}}}$ is the pedigree relationship matrix with metafounders, ${\mathbf{G}}^{0.5}$ is the genomic realized relationship matrix with allele frequencies equal to 0.5; subscripts 1 and 2 stand for non-genotyped and genotyped animals, respectively.

The construction of the relationship matrix ${\mathbf{A}}^{{\varvec{\Gamma}}}$ is based on the estimated metafounder relationship matrix, ${\varvec{\Gamma}}$ [17], which represents the within- and across-population relationship matrix and is expressed as follows:

$${\varvec{\Gamma}}=\left[\begin{array}{cc}{\upgamma }_{\mathrm{B}}& {\upgamma }_{\mathrm{B},\mathrm{H}}\\ \mathrm{sym}& {\upgamma }_{\mathrm{H}}\end{array}\right],$$

where ${\upgamma }_{\mathrm{B}}$ is the metafounder relationship for BBL; ${\upgamma }_{\mathrm{H}}$ is the metafounder relationship for HOL; ${\upgamma }_{\mathrm{B},\mathrm{H}}$ is the across-metafounder relationship between the BBL and HOL populations. The generalized least squares method was used to estimate ${\varvec{\Gamma}}$ as described by Garcia-Baccino et al. [28].

The relationship matrix ${\mathbf{G}}^{0.5}$ is constructed as:

$${\mathbf{G}}^{0.5}=\frac{\mathbf{Z}\mathbf{Z}\mathbf{^{\prime}}}{\mathrm{s}},$$

where $\mathbf{Z}$ is the incidence matrix with elements of 1, 0 and − 1 for *AA*, *Aa*, and *aa*, respectively; $\mathrm{s}=\mathrm{m}/2$.

The genetic variance and covariance parameters from MF-ssGBLUP were estimated under the assumption that founders are related, while in other models usually unrelated founders are assumed for the genetic variance. To be comparable with estimates from other models that estimate genetic variance for unrelated founders, such as standard ssGBLUP and BS-ssGBLUP in our study, we multiplied the estimates of the genetic parameters estimated with MF-ssGBLUP by $1+\frac{\overline{\mathrm{diag }({\varvec{\Gamma}})}}{2}-\overline{{\varvec{\Gamma}} }$, following the suggestion of Legarra et al. [17].

### BS-ssGBLUP

BS-ssGBLUP assumes that the substitution effects of breed-specific alleles differ between breeds. This method was developed by Christensen et al. [13] based on previous studies [14, 29].

The statistical bivariate model for BS-ssGBLUP is:

3 $$\mathbf{y}=\mathbf{X}\mathbf{b}+{\mathbf{Z}}_{\mathbf{B}}{\mathbf{u}}_{\mathbf{B}}+{\mathbf{Z}}_{\mathbf{H}}{\mathbf{u}}_{\mathbf{H}}+\mathbf{e},$$

where ${\mathbf{y}}$, $\mathbf{b}$, $\mathbf{e}$, and $\mathbf{X}$ are as defined above; ${\mathbf{u}}_{\mathbf{B}}$ is the vector of random additive genetic effects from BBL for ADG and FCR, ${\mathbf{u}}_{\mathbf{H}}$ is the vector of random additive genetic effects from HOL for ADG and FCR; ${\mathbf{Z}}_{\mathbf{B}}$ and ${\mathbf{Z}}_{\mathbf{H}}$ are the corresponding incidence matrices.

It is assumed that the random effects follow normal distributions, i.e. ${\mathbf{u}}_{\mathbf{B}}\sim \mathrm{N}(\boldsymbol{0}, {\sum }_{{\mathbf{u}}_{\mathbf{B}}}\otimes {\mathbf{H}}_{\mathbf{B}})$ and ${\mathbf{u}}_{\mathbf{H}}\sim \mathrm{N}(\boldsymbol{0}, {\sum }_{{\mathbf{u}}_{\mathbf{H}}}\otimes {\mathbf{H}}_{\mathbf{H}})$, where ${\mathbf{H}}_{\mathbf{B}}$ and ${\mathbf{H}}_{\mathbf{H}}$ are combined pedigree-based and marker-based breed specific partial relationship matrices for BBL and HOL; ${\sum }_{{\mathbf{u}}_{\mathbf{B}}}$ is the genetic (co)variance matrix for BBL, ${\sum }_{{\mathbf{u}}_{\mathbf{H}}}$ is the genetic (co)variance matrix for HOL. The (co)variance matrices are as follows:

$${\sum }_{{\mathbf{u}}_{\mathbf{B}}}=\left[\begin{array}{cc}{\upsigma }_{{{\mathrm{u}}_{\mathrm{B}}}_{\mathrm{ADG}}}^{2}& {\upsigma }_{{{\mathrm{u}}_{\mathrm{B}}}_{\mathrm{ADG}}{{\mathrm{u}}_{\mathrm{B}}}_{\mathrm{FCR}}}\\ \mathrm{sym}& {\upsigma }_{{{\mathrm{u}}_{\mathrm{B}}}_{\mathrm{FCR}}}^{2}\end{array}\right] ,$$

$${\mathrm{and }\sum }_{{\mathbf{u}}_{\mathbf{H}}}=\left[\begin{array}{cc}{\upsigma }_{{{\mathrm{u}}_{\mathrm{H}}}_{\mathrm{ADG}}}^{2}& {\upsigma }_{{{\mathrm{u}}_{\mathrm{H}}}_{\mathrm{ADG}}{{\mathrm{u}}_{\mathrm{H}}}_{\mathrm{FCR}}}\\ \mathrm{sym}& {\upsigma }_{{{\mathrm{u}}_{\mathrm{H}}}_{\mathrm{FCR}}}^{2}\end{array}\right],$$

where ${\upsigma }_{{{\mathrm{u}}_{\mathrm{B}}}_{\mathrm{ADG}}}^{2}$ is the additive genetic variance of ADG from BBL, ${\upsigma }_{{{\mathrm{u}}_{\mathrm{B}}}_{\mathrm{FCR}}}^{2}$ is the additive genetic variance of FCR from BBL, ${\upsigma }_{{{\mathrm{u}}_{\mathrm{B}}}_{\mathrm{ADG}}{{\mathrm{u}}_{\mathrm{B}}}_{\mathrm{FCR}}}$ is the additive genetic covariance between ADG and FCR from BBL; ${\upsigma }_{{{\mathrm{u}}_{\mathrm{H}}}_{\mathrm{ADG}}}^{2}$ is the additive genetic variance of ADG from HOL, ${\upsigma }_{{{\mathrm{u}}_{\mathrm{H}}}_{\mathrm{FCR}}}^{2}$ is the additive genetic variance of FCR from breed HOL, ${\upsigma }_{{{\mathrm{u}}_{\mathrm{H}}}_{\mathrm{ADG}}{{\mathrm{u}}_{\mathrm{H}}}_{\mathrm{FCR}}}$ is the additive genetic covariance between ADG and FCR from HOL.

The breed-specific matrix ${\mathbf{H}}_{\mathbf{B}}$ is defined as:

$${\mathbf{H}}_{\mathbf{B}}=\left[\begin{array}{cc}{\mathbf{A}}_{\boldsymbol{11}}^{(\mathbf{B})}-{\mathbf{A}}_{\boldsymbol{12}}^{(\mathbf{B})}{{\mathbf{A}}_{\boldsymbol{22}}^{(\mathbf{B})}}^{-1}{\mathbf{A}}_{\boldsymbol{21}}^{(\mathbf{B})}+{\mathbf{A}}_{\boldsymbol{12}}^{(\mathbf{B})}{{\mathbf{A}}_{\boldsymbol{22}}^{(\mathbf{B})}}^{-1}{\mathbf{G}}^{(\mathbf{B})}{{\mathbf{A}}_{\boldsymbol{22}}^{(\mathbf{B})}}^{-\boldsymbol{1}}{\mathbf{A}}_{\boldsymbol{21}}^{(\mathbf{B})}& {\mathbf{A}}_{\boldsymbol{12}}^{(\mathbf{B})}{{\mathbf{A}}_{\boldsymbol{22}}^{(\mathbf{B})}}^{-\boldsymbol{1}}{\mathbf{G}}^{(\mathbf{B})}\\ {\mathbf{G}}^{(\mathbf{B})}{{\mathbf{A}}_{\boldsymbol{22}}^{(\mathbf{B})}}^{-\boldsymbol{1}}{\mathbf{A}}_{\boldsymbol{21}}^{(\mathbf{B})}& (1-\upomega ){\mathbf{G}}^{(\mathbf{B})}+\upomega {{\mathbf{A}}_{\boldsymbol{22}}^{(\mathbf{B})}}^{-1}\end{array}\right],$$

where $\upomega$ is as defined above; ${\mathbf{A}}^{(\mathbf{B})}$ is the breed-specific pedigree relationship matrix from BBL, ${\mathbf{G}}^{(\mathbf{B})}$ is the breed-specific genomic realized relationship matrix from BBL; subscripts 1 and 2 stand for non-genotyped and genotyped animals, respectively.

The breed-specific pedigree relationship matrix ${\mathbf{A}}^{(\mathbf{B})}$ was previously described by García-Cortés and Toro [14]. Matrix ${\mathbf{G}}^{(\mathbf{B})}$ is split into submatrices with indices denoting genotyped BBL and crossbred animals as follows:

$${\mathbf{G}}^{(\mathbf{B})}=\left[\begin{array}{cc}{\mathbf{G}}_{\mathbf{B},\mathbf{B}}^{(\mathbf{B})}& {\mathbf{G}}_{\mathbf{B},\mathbf{B}\mathbf{H}}^{(\mathbf{B})}\\ \mathbf{s}\mathbf{y}\mathbf{m}& {\mathbf{G}}_{\mathbf{B}\mathbf{H},\mathbf{B}\mathbf{H}}^{(\mathbf{B})}\end{array}\right],$$

with these submatrices being defined as:

$${\mathbf{G}}_{\mathbf{B},\mathbf{B}}^{(\mathbf{B})}=\frac{({\mathbf{M}}_{\mathbf{B}}-2{\mathbf{p}}_{\mathbf{B}}\boldsymbol{1}){({\mathbf{M}}_{\mathbf{B}}-\boldsymbol{2}{\mathbf{p}}_{\mathbf{B}}\boldsymbol{1})}^{^{\prime}}}{2{\mathbf{p}}_{\mathbf{B}}^{^{\prime}}(\boldsymbol{1}-{\mathbf{p}}_{\mathbf{B}})},$$

$${\mathbf{G}}_{\mathbf{B},\mathbf{B}\mathbf{H}}^{(\mathbf{B})}=\frac{({\mathbf{M}}_{\mathbf{B}}-\boldsymbol{2}{\mathbf{p}}_{\mathbf{B}}\boldsymbol{1}){({\mathbf{Q}}_{\mathbf{B}}-{\mathbf{p}}_{\mathbf{B}}\boldsymbol{1})}^{^{\prime}} }{2{\mathbf{p}}_{\mathbf{B}}^{^{\prime}}(\boldsymbol{1}-{\mathbf{p}}_{\mathbf{B}})},$$

$$\mathrm{and }\,{\mathbf{G}}_{\mathbf{B}\mathbf{H},\mathbf{B}\mathbf{H}}^{(\mathbf{B})}=\frac{({\mathbf{Q}}_{\mathbf{B}}-{\mathbf{p}}_{\mathbf{B}}\boldsymbol{1}){({\mathbf{Q}}_{\mathbf{B}}-{\mathbf{p}}_{\mathbf{B}}\boldsymbol{1})}^{^{\prime}}}{2{\mathbf{p}}_{\mathbf{B}}^{^{\prime}}(\boldsymbol{1}-{\mathbf{p}}_{\mathbf{B}})},$$

where ${\mathbf{M}}_{\mathbf{B}}$ and ${\mathbf{Q}}_{\mathbf{B}}$ contain the breed BBL specific allele contents of the reference allele for BBL (coded as 0, 1, or 2) and for BH (coded as 0 or 1), respectively, for which tracing of the breed of origin of alleles is required; $\boldsymbol{1}$ is a vector of 1s; and ${\mathbf{p}}_{\mathbf{B}}$ is the vector of BBL specific allele frequencies. Finally, matrix ${\mathbf{G}}^{(\mathbf{B})}$ is adjusted to be compatible with matrix ${\mathbf{A}}^{(\mathbf{B})}$, as described by Christensen et al. [13]. The definition of the breed-specific matrix ${\mathbf{H}}_{\mathbf{H}}$ is similar to the definition of the ${\mathbf{H}}_{\mathbf{B}}$ matrix.

Tracing the breed of origin of alleles in F1 crosses is expected to be very accurate [30], and was conducted on the phased genotypes, separately, for each chromosome per individual. Among the 1780 genotyped crossbred animals, 1447 crossbred animals had 47 genotyped sires, whereas for the 333 remaining crossbred animals, none of the parents were genotyped. When the sire (or dam) was genotyped, four comparisons between crossbred and purebred phased alleles were made. For each comparison, when a crossbred allele differed from the corresponding purebred allele, it was counted as a difference. The chromosome with the smallest number of differences was assigned to the breed of the parent. When neither of the parents was genotyped, for each non-overlapping sliding window of 50 consecutive SNPs, comparisons between the two crossbred segments of phased alleles and segments of phased alleles in the reference panel were made for each breed. For each of the two crossbred segments, the number of copies was counted for each breed, and the segment was considered to originate from the breed with the largest number of copies. Finally, each crossbred chromosome was assigned to the breed from which the majority of its segments originated. This procedure is the same as in Xiang et al. [5].

For tracing of alleles and the construction of the breed-specific matrices ${\mathbf{H}}_{\mathbf{B}}$ and ${\mathbf{H}}_{\mathbf{H}}$ in BS-SSGBLUP, we developed an R package named cBar2, which has been uploaded on github (https://github.com/TXiang-lab/cBar2).

Estimation of genetic parameters in the above bivariate models with the three methods was carried out using the restricted maximum likelihood (REML) algorithm in the software DMU [31] via the wrapper of R package blupADC [32].

The heritability and genetic correlation estimates and their standard errors in ssGBLUP and MF-ssGBLUP were calculated as described by Falconer [33] and Mrode [34]. For BS-ssGBLUP, the heritability estimates for ADG and FCR were calculated as ${\mathrm{h}}_{\mathrm{ADG}}^{2}=\frac{{\upsigma }_{{{\mathrm{u}}_{\mathrm{BH}}}_{\mathrm{ADG}}}^{2}}{{\upsigma }_{{{\mathrm{u}}_{\mathrm{BH}}}_{\mathrm{ADG}}}^{2}+{\upsigma }_{{\mathrm{e}}_{\mathrm{ADG}}}^{2}}$ and ${\mathrm{h}}_{\mathrm{FCR}}^{2}=\frac{{\upsigma }_{{{\mathrm{u}}_{\mathrm{BH}}}_{\mathrm{FCR}}}^{2}}{{\upsigma }_{{{\mathrm{u}}_{\mathrm{BH}}}_{\mathrm{FCR}}}^{2}+{\upsigma }_{{\mathrm{e}}_{\mathrm{FCR}}}^{2}}$, where ${\upsigma }_{{{\mathrm{u}}_{\mathrm{BH}}}_{\mathrm{ADG}}}^{2}$ and ${\upsigma }_{{{\mathrm{u}}_{\mathrm{BH}}}_{\mathrm{FCR}}}^{2}$ are defined as $0.5\left({\upsigma }_{{{\mathrm{u}}_{\mathrm{B}}}_{\mathrm{ADG}}}^{2}+{\upsigma }_{{{\mathrm{u}}_{\mathrm{H}}}_{\mathrm{ADG}}}^{2}\right)$ and $0.5\left({\upsigma }_{{{\mathrm{u}}_{\mathrm{B}}}_{\mathrm{FCR}}}^{2}+{\upsigma }_{{{\mathrm{u}}_{\mathrm{H}}}_{\mathrm{FCR}}}^{2}\right)$. The standard errors of the heritabilities, ${\upsigma }_{\left({\mathrm{h}}_{\mathrm{ADG}}^{2}\right)}$ and ${\upsigma }_{\left({\mathrm{h}}_{\mathrm{FCR}}^{2}\right)}$, were obtained by the deltaMethod implemented in the R package msm [35]. The genetic correlation between ADG and FCR was calculated as $\mathrm{r}=\frac{0.5({\upsigma }_{{{\mathrm{u}}_{\mathrm{B}}}_{\mathrm{ADG}}{{\mathrm{u}}_{\mathrm{B}}}_{\mathrm{FCR}}}+{\upsigma }_{{{\mathrm{u}}_{\mathrm{H}}}_{\mathrm{ADG}}{{\mathrm{u}}_{\mathrm{H}}}_{\mathrm{FCR}}})}{\sqrt{{\upsigma }_{{{\mathrm{u}}_{\mathrm{BH}}}_{\mathrm{ADG}}}^{2}*{\upsigma }_{{{\mathrm{u}}_{\mathrm{BH}}}_{\mathrm{FCR}}}^{2}}}$, and its standard error was also obtained by the deltaMethod.

### Model-based reliability

For ssGBLUP, the model-based reliability was calculated as follows:

$${\mathrm{Rel}}_{\mathrm{i}}=\boldsymbol{1}-\frac{{\mathbf{P}\mathbf{E}\mathbf{V}}_{\mathrm{i},\mathrm{i}}}{{\mathbf{H}}_{\mathbf{i},\mathbf{i}}{\upsigma }_{\mathrm{u}}^{2}},$$

where $\mathbf{H}$ is as defined previously; ${\mathrm{Rel}}_{\mathrm{i}}$ is the reliability of the individual $\mathrm{i}$, ${\upsigma }_{\mathrm{u}}^{2}$ is the additive genetic variance estimated with BS-ssGBLUP, $\mathbf{P}\mathbf{E}\mathbf{V}$ is the prediction error (co)variance matrix, which can be obtained by inverting the coefficient matrix of Henderson’s mixed model equations corresponding to the model used [34].

Both MF-ssGBLUP and BS-ssGBLUP can model the genetic difference between breeds, and the individual model-based reliability can be calculated within each breed [13, 36]. For MF-ssGBLUP, the model-based reliability was calculated as described in Bermann et al. [36].

For individual $\mathrm{i}$, the reliability within each metafounder is calculated as:

$${\mathrm{Rel}}_{\mathrm{i}}^{\mathrm{mf}}=\boldsymbol{1}-\frac{\mathbf{P}\mathbf{E}\mathbf{V}\left({\mathbf{u}}_{\mathrm{i}}\right)+\mathbf{P}\mathbf{E}\mathbf{V}\left({\mathbf{u}}_{\mathrm{mf}}\right)-2\mathbf{P}\mathbf{E}\mathbf{V}\left({\mathbf{u}}_{\mathrm{i}}, {\mathbf{u}}_{\mathbf{m}\mathbf{f}}\right)}{\left({\mathbf{H}}_{\mathbf{i}\mathbf{i}}^{\left({\varvec{\Gamma}}\right)}+{\mathbf{H}}_{\mathbf{m}\mathbf{f},\mathbf{m}\mathbf{f}}^{\left({\varvec{\Gamma}}\right)}-2{\mathbf{H}}_{\mathbf{i},\mathbf{m}\mathbf{f}}^{\left({\varvec{\Gamma}}\right)}\right){\upsigma }_{\mathrm{u}\left(\Gamma \right)}^{2}},$$

where $\mathbf{u}$, $\mathbf{P}\mathbf{E}\mathbf{V}$ and ${\mathbf{H}}^{{\varvec{\Gamma}}}$ are as defined previously; ${\mathrm{Rel}}_{\mathrm{i}}^{\mathrm{mf}}$ is the reliability of individual $\mathrm{i}$ within metafounder $\mathrm{mf}$; and ${\upsigma }_{\mathrm{u}(\Gamma )}^{2}$ is the additive genetic variance estimated with MF-ssGBLUP.

For BS-ssGBLUP, the model-based reliability is calculated as follows:

$${\mathrm{Rel}}_{\mathrm{i}}=\boldsymbol{1}-\frac{{\mathbf{P}\mathbf{E}\mathbf{V}}_{\mathrm{i},\mathrm{i}}}{{\mathbf{H}}_{{\mathbf{A}}_{\mathbf{i},\mathbf{i}}}{\upsigma }_{\mathrm{u}}^{2}},$$

where ${\mathbf{H}}_{\mathbf{A}}$ and $\mathbf{P}\mathbf{E}\mathbf{V}$ are as defined previously; ${\mathrm{Rel}}_{\mathrm{i}}$ is the reliability of the individual $\mathrm{i}$, and ${\upsigma }_{\mathrm{u}}^{2}$ is the additive genetic variance estimated with BS-ssGBLUP.

In this study, we investigated the model-based reliabilities of BBL sires having offspring with phenotypes.

### Estimators of the LR method

In this study, four estimators, i.e. bias ($\widehat{\Delta }$), dispersion ($\widehat{\mathrm{b}}$), population accuracy ($\widehat{\mathrm{acc}}$) and ratio of accuracies ($\widehat{\uprho }$) were estimated with the LR method [37] and were used to evaluate the impact of each of the three methods (ssGBLUP, MF-ssGBLUP, and BS-ssGBLUP) on the estimated breeding values (EBV) for crossbred performance, since the LR method has proven to show better analytical properties than the ordinary cross-validation method [37–39]. EBV of focal individuals were denoted as ${\widehat{\mathbf{u}}}_{\mathbf{p}}$ and ${\widehat{\mathbf{u}}}_{\mathbf{w}}$ based on the partial and the whole dataset, respectively. The partial dataset was defined as the set of crossbred animals which were born before a specified cut-off date (we used two cut-off dates in this study, April 1 2021 and May 1 2021), and focal individuals were those born after the specific cut-off date. The number of individuals in the partial datasets for each trait are in Table 1. For BS-ssGBLUP, EBV are equal to the sum of ${\mathbf{u}}_{\mathbf{H}}$ and ${\mathbf{u}}_{\mathbf{B}}$. The estimators are summarized below.

### Bias

The bias estimator $\widehat{\Delta }$ is defined as the difference between the mean of EBV based on the partial dataset and the mean of EBV based on the whole dataset, i.e. $\widehat{\Delta } =\overline{{\widehat{\mathbf{u}} }_{\mathbf{p}}}-\overline{{\widehat{\mathbf{u}} }_{\mathbf{w}}}$.

In absence of bias, the expected value of this estimator is 0.

### Dispersion

The dispersion estimator is defined as the slope of the regression of ${\widehat{\mathbf{u}}}_{\mathbf{w}}$ on ${\widehat{\mathbf{u}}}_{\mathbf{p}}$, which is equal to $\widehat{\mathrm{b}}=\frac{\mathrm{Cov}({\widehat{\mathbf{u}}}_{\mathbf{w}},{\widehat{\mathbf{u}}}_{\mathbf{p}})}{\mathrm{Var}({\widehat{\mathbf{u}}}_{\mathbf{p}})}$. The expected value of this estimator is 1 under the assumption that ${\widehat{\mathbf{u}}}_{\mathbf{p}}$ has no dispersion bias, while $\widehat{\mathrm{b}}$< 1 indicates over-dispersion, and $\widehat{\mathrm{b}}$> 1 indicates under-dispersion of ${\widehat{\mathbf{u}}}_{\mathbf{p}}$.

### Population accuracy

The population accuracy of focal individuals based on the partial dataset can be calculated as $\widehat{\mathrm{acc}}=\sqrt{\frac{\mathrm{Cov}({\widehat{\mathbf{u}}}_{\mathbf{w}},{\widehat{\mathbf{u}}}_{\mathbf{p}})}{(1+\overline{\mathrm{F} }-2\overline{\mathrm{f} } ){\upsigma }_{\mathrm{u},\infty }^{2}}}$, where $\overline{\mathrm{F} }$ is the average inbreeding coefficient of focal individuals, 2 $\overline{\mathrm{f} }$ is the average relationship between focal individuals, and ${\upsigma }_{\mathrm{u},\infty }^{2}$ is the estimated genetic variance with a partial dataset (assuming that the focal individuals are not under selection in the partial dataset).

### Ratio of population accuracies

The ratio of population accuracies estimator is defined as the Pearson correlation between ${\widehat{\mathbf{u}}}_{\mathbf{w}}$ and ${\widehat{\mathbf{u}}}_{\mathbf{p}}$, which is equal to $\widehat{\uprho }=\frac{\mathrm{Cov}({\widehat{\mathbf{u}}}_{\mathbf{w}},{\widehat{\mathbf{u}}}_{\mathbf{p}})}{\sqrt{\mathrm{Var}({\widehat{\mathbf{u}}}_{\mathbf{w}})\mathrm{Var}({\widehat{\mathbf{u}}}_{\mathbf{p}})}}$. This is an estimator for $\frac{{\mathrm{acc}}_{\mathrm{p}}}{{\mathrm{acc}}_{\mathrm{w}}}$, where ${\mathrm{acc}}_{\mathrm{p}}$ is the population accuracy based on the partial dataset, and ${\mathrm{acc}}_{\mathrm{w}}$ is the population accuracy based on the whole dataset.

## Results

### Genetic parameters

Estimated variance components and heritabilities for ADG and FCR and the estimated genetic correlations between ADG and FCR are in Table 2. The genetic variances and covariance obtained with MF-ssGBLUP were scaled for comparison with those of the other two methods. For MF-ssGBLUP, the metafounder relationship coefficients ${\upgamma }_{\mathrm{B}}$, ${\upgamma }_{\mathrm{BH}}$ and ${\upgamma }_{\mathrm{H}}$ were estimated to be 0.702, 0.570, and 0.672, respectively.

**Table 2:** Estimates of variance components and their standard error (SE) obtained with three methods

Trait	Method	${{\varvec{\upsigma}}}_{\mathbf{u}}^{2}$ (SE)	${{\varvec{\upsigma}}}_{\mathbf{B}}^{2}$ (SE)	${{\varvec{\upsigma}}}_{\mathbf{H}}^{2}$ (SE)	${{\varvec{\upsigma}}}_{\mathbf{e}}^{2}$ (SE)	${\mathbf{r}}_{\mathbf{g}}$ (SE)	${\mathbf{h}}^{2}$ (SE)
ADG	ssGBLUP	0.008(0.004)	–	–	0.090(0.004)	− 0.531(0.239)	0.082(0.041)
	MF-ssGBLUP	0.007(0.004)	–	–	0.085(0.004)	− 0.515(0.251)	0.076(0.041)
	BS-ssGBLUP	–	0.004(0.003)	0.023(0.009)	0.083(0.005)	− 0.620(0.197)	0.140(0.050)
FCR	ssGBLUP	0.189(0.093)	–	–	2.191(0.099)	− 0.531(0.239)	0.079(0.038)
	MF-ssGBLUP	0.192(0.096)	–	–	2.199(0.097)	− 0.515(0.251)	0.080(0.039)
	BS-ssGBLUP	–	0.227(0.119)	0.106(0.201)	2.215(0.123)	− 0.620(0.197)	0.070(0.048)

${\upsigma }_{\mathrm{u}}^{2}$: additive genetic variance; ${\upsigma }_{\mathrm{B}}^{2}$: additive genetic variance for breed BBL; ${\upsigma }_{\mathrm{H}}^{2}$: additive genetic variance for breed HOL; ${\upsigma }_{\mathrm{e}}^{2}$: residual variance; ${\mathrm{r}}_{\mathrm{g}}$: genetic correlation between ADG and FCR; ${\mathrm{h}}^{2}$: heritability, ${\mathrm{h}}^{2}=\frac{{\upsigma }_{\mathrm{u}}^{2}}{{\upsigma }_{\mathrm{u}}^{2}+{\upsigma }_{\mathrm{e}}^{2}}$ for ssGBLUP and MF-ssGBLUP, ${\mathrm{h}}^{2}=\frac{0.5({\upsigma }_{\mathrm{B}}^{2}+{\upsigma }_{\mathrm{H}}^{2})}{0.5({\upsigma }_{\mathrm{B}}^{2}+{\upsigma }_{\mathrm{H}}^{2}+{\upsigma }_{\mathrm{e}}^{2})}$ for BS-ssGBLUP

MF-ssGBLUP: Metafounder ssGBLUP; BS-ssGBLUP: Breed-specific ssGBLUP; ADG: average daily gain; FCR: feed conversion ratio

The estimated variance components for ssGBLUP and MF-ssGBLUP were similar. The estimates of the heritabilities for ssGBLUP and MF-ssGBLUP were also similar for both ADG (0.082 and 0.076) and FCR (0.079 and 0.080). However, for BS-ssGBLUP, ADG had a heritability estimate of 0.140, which differed from the estimate obtained with the other two methods. The genetic correlation between ADG and FCR was negative and moderate to high for all methods, i.e. − 0.531(0.239), − 0.515(0.251), and − 0.620(0.197) for ssGBLUP, MF-ssGBLUP and BS-ssGBLUP, respectively.

### Model-based reliability

Table 3 shows the mean model-based reliabilities of purebred sires for their crossbred performance for ssGBLUP, MF-ssGBLUP and BS-ssGBLUP. Model-based reliabilities were computed for sires having offspring with phenotypes, and are presented as an average of all sires, an average of genotyped sires, and an average of non-genotyped sires. Among these 67 sires, only 47 have been genotyped. On average, genotyped sires had higher reliabilities than non-genotyped sires, regardless of which method was used. For ADG, MF-ssGBLUP had the highest model-based reliability (0.323), and BS-ssGBLUP had the lowest model-based reliability (0.221), while for FCR, BS-ssGBLUP and MF-ssGBLUP had the highest model-based reliability (0.348), and ssGBLUP had the lowest model-based reliability (0.261). For both traits, MF-ssGBLUP always had a higher model-based reliability than ssGBLUP.

**Table 3:** Mean model-based reliability of purebred bulls for their crossbred performance

Trait	Method	All	Genotyped	Non-genotyped
ADG	ssGBLUP	0.243	0.266	0.188
	MF-ssGBLUP	0.323	0.354	0.251
	BS-ssGBLUP	0.221	0.243	0.170
FCR	ssGBLUP	0.261	0.285	0.206
	MF-ssGBLUP	0.348	0.380	0.274
	BS-ssGBLUP	0.348	0.374	0.287

MF-ssGBLUP: Metafounder ssGBLUP; BS-ssGBLUP: Breed-specific ssGBLUP; ADG: average daily gain; FCR: feed conversion ratio

### Predictive ability

Four estimators ($\widehat{\Delta }$, $\widehat{\mathrm{b}}$, $\widehat{\mathrm{acc}}$ and $\widehat{\uprho }$) in the LR method were used to evaluate the predictive ability of ssGBLUP, MF-ssGBLUP, and BS-ssGBLUP for two focal sets of individuals. The results for the different datasets of individuals are in Table 4 for those with a cut-off date at April 1 2021 and in Additional file 2: Table S1 for those with a cut-off date at May 1 2021. The results differed slightly between datasets, but the conclusions were similar. Therefore, in the remainder of the paper, we focus only on the results in Table 4.

**Table 4:** Bias ($\widehat{\Delta }$), dispersion ($\widehat{\mathbf{b}}$), population accuracy ($\widehat{\mathbf{a}\mathbf{c}\mathbf{c}}$) and ratio of population accuracies ($\widehat{{\varvec{\uprho}}}$) of EBV for focal individuals (cut-off date, April 1, 2021) obtained with three methods

Trait	Method	$\widehat{\Delta }$	$\widehat{\mathbf{b}}$	$\widehat{\mathbf{a}\mathbf{c}\mathbf{c}}$	$\widehat{{\varvec{\uprho}}}$
ADG	ssGBLUP	0.009	1.035	0.267	0.714
	MF-ssGBLUP	0.011	1.030	0.273	0.729
	BS-ssGBLUP	0.006	1.015	0.239	0.520
FCR	ssGBLUP	− 0.036	0.782	0.210	0.691
	MF-ssGBLUP	0.003	0.786	0.213	0.699
	BS-ssGBLUP	0.001	0.787	0.257	0.737

MF-ssGBLUP: Metafounder ssGBLUP; BS-ssGBLUP: Breed-specific ssGBLUP; ADG: average daily gain; FCR: feed conversion ratio

As shown in Table 4, the differences between $\widehat{\Delta }$ across the three methods were small. For all methods, the values of $\widehat{\Delta }$ were close to the expected value (equal to 0) for both traits, while the values of $\widehat{\mathrm{b}}$ were close to the expected value (equal to 1) for ADG and deviated from the expected value for FCR. For ADG, population accuracy ($\widehat{\mathrm{acc}}$) was highest (0.273) with MF-ssGBLUP, and lowest (0.239) with BS-ssGBLUP, while for FCR, it was highest (0.257) with BS-ssGBLUP, and lowest (0.210) with ssGBLUP. For both traits, population accuracy was higher with MF-ssGBLUP than with ssGBLUP. The ratios of population accuracies based on the partial and whole datasets were for ssGBLUP, MF-ssGBLUP and BS-ssGBLUP, respectively, 0.714, 0.729, and 0.520 for ADG, and 0.691, 0.699, and 0.737 for FCR.

## Discussion

In this work, first we compared the estimates of genetic parameters for ADG and FCR obtained with ssGBLUP, MF-ssGBLUP and BS-ssGBLUP. In general, variance components and heritability estimates for FCR did not differ considerably between methods, while for ADG, those estimated with BS-ssGBLUP deviated largely from those estimated with ssGBLUP and MF-ssGBLUP. Then, we evaluated the impact of these methods on the predictive ability for crossbred performance. For both traits, the estimators ($\widehat{\Delta }$, $\widehat{\mathrm{b}}$, and $\widehat{\mathrm{acc}}$) in the LR method showed that the predictive ability of MF-ssGBLUP was always superior to that of ssGBLUP, whereas the comparison of the predictive ability of BS-ssGBLUP with the other two methods showed no consistent result.

### Genetic parameters

Variance components, heritabilities and genetic correlations obtained with ssGBLUP and with MF-ssGBLUP were similar for both ADG and FCR. This observation is in line with previous studies [18, 40]. However, for BS-ssGBLUP, the estimated genetic parameters for FCR were similar to those with the other two methods, while the result was opposite for ADG. As shown in Table 2, the additive genetic variance for FCR in the sire breed and dam breed was 0.227 and 0.106, respectively, while for ADG, it was 0.004, and 0.023, respectively. Our results are not consistent with those reported by Poulsen et al. [19] on simulated data, who found that the estimated variance components from the three methods were similar, with those from MF-ssGBLUP being closer to those from BS-ssGBLUP than those from ssGBLUP. One possible reason for this difference may be the lack of sufficient information in our dataset to distinguish the additive genetic variances between the sire breed and the dam breed in BS-ssGBLUP. To date, few studies have examined whether there are differences in the variance components, heritabilities and genetic correlations between these three methods, and further investigation is needed.

In our study, ADG and FCR were lowly heritable with heritability estimates ranging from 0.081 to 0.153 for ADG, and from 0.080 to 0.084 for FCR. A few studies have reported similarly low values [41–43], but in general, ADG and FCR are considered as moderately to highly heritable traits [44, 45]. Our results could be due to the short testing period used. In general, ADG and FCR are normally collected over longer test periods (3–6 months) [44, 45] than the one-month test period in our study. Furthermore, Ahlberg et al. [46] pointed out that during different periods, the phenotypic correlations for each shortened test duration differed. Although the heritability estimates for ADG and FCR are lower than those reported in previous studies, the moderate to high negative genetic correlation between ADG and FCR is in agreement with other studies [44, 45].

### Model-based reliabilities

In terms of model-based reliability with MF-ssGBLUP, the usual definition (expressed as 1 $-\frac{{\mathrm{PEV}}_{\mathrm{i},\mathrm{i}}}{{\mathrm{H}}_{\mathrm{ii}}^{\left(\Gamma \right) }{\upsigma }_{\mathrm{u}\left(\Gamma \right)}^{2}}$) is inappropriate for metafounder relationships, as pointed out by Bermann et al. [36], since it would underestimate reliabilities. To account for this, Bermann et al. [36] proposed a new method where reliabilities are calculated from contrasts to a reference metafounder. By applying this method with MF-ssGBLUP in our study, the reliabilities of purebred sires increased by almost 30%, compared with the usual definition (results not shown). In our study, there were two metafounders, one representing BBL and the other HOL. Each individual would have two reliabilities corresponding to BBL and HOL. For BS-ssGBLUP, there were also two reliabilities based on two breed-specific relationship matrices.

Within each method, for ADG and FCR, the reliabilities for the genotyped sire group were always larger than for the non-genotyped sire group. This result is in line with previous studies [5, 18]. In terms of reliabilities across methods, as expected, MF-ssGBLUP always had higher reliabilities than ssGBLUP. However, for BS-ssGBLUP, the results were not consistent, i.e. for FCR the reliabilities from BS-ssGBLUP were similar to those from MF-ssGBLUP, but for ADG they were the lowest among the three methods. This could be due to the fact that the genetic parameters estimated for ADG with BS-ssGBLUP deviated a lot from the estimated parameters with the other two methods, but also to the small sample size for the sires.

### Predictive ability

In this study, four estimators, $\widehat{\Delta }$, $\widehat{\mathrm{b}}$, $\widehat{\mathrm{acc}}$ and $\widehat{\uprho }$, in the LR method [37] were used to evaluate the predictive ability of ssGBLUP, BS-ssGBLUP and MF-ssGBLUP. Table 4 shows that for $\widehat{\Delta }$ and $\widehat{\mathrm{b}}$, there are little differences between these three methods.

The difference between $\widehat{\mathrm{b}}$ and its expected value showed that the EBV of FCR were over-dispersed, and that their deviation from the expected value were larger than for ADG. Mäntysaari et al. [47] have suggested that over-dispersion of EBV may be due to strong selection. In terms of $\widehat{\uprho }$, Legarra and Reverter [37] pointed out that it is an estimator of change in population accuracy, but not a measure of population accuracy. Its reciprocal minus 1 can be interpreted as the relative increase of population accuracy from partial to whole information. For example, a value of 0.699 for the $\widehat{\uprho }$ of FCR with MF-ssGBLUP means that the corresponding increase in population accuracy from the partial to the whole dataset is 43.1%.

As expected, MF-ssGBLUP always had a slightly higher population accuracy than ssGBLUP. In a multiple-breed beef cattle population, Junqueira et al. [20] and Kluska et al. [21] found that, compared to ssGBLUP, MF-ssGBLUP decreased bias in genomic evaluations. The same result has also been found for crossbred pigs [18]. However, with BS-ssGBLUP, opposite $\widehat{\mathrm{acc}}$ values were obtained for ADG and FCR, which is similar to the model-based reliabilities that also showed opposite results for ADG and FCR with BS-ssGBLUP. As already mentioned, one possible reason is that the estimated genetic variance with BS-ssGBLUP for ADG deviated a lot from the estimated parameters with the other two methods. For FCR, for which the estimated genetic variance components were similar across the three methods, both BS-ssGBLUP and MF-ssGBLUP had a better predictive ability than ssGBLUP, which is in line with a previous study [19]. In addition, we found that for FCR, BS-ssGBLUP had a better predictive ability than MF-ssGBLUP, which was not consistent with the results of Poulsen et al. [19] who reported similar predictive abilities for BS-ssGBLUP and MF-ssGBLUP. A possible reason for the conflicting results observed in our study may be that the metafounder relationship matrix ${\varvec{\Gamma}}$ could be accurately estimated in the simulated dataset in Poulsen et al. [19], whereas in our case the estimates of ${\varvec{\Gamma}}$ maybe inaccurate, and could be biased because of the small number of genotyped animals, as is the case for BBL. Inaccurate estimates of **Γ** may affect the performance of MF-ssGBLUP. Moreover, missing genotypes were imputed based on a combination of different SNP panels (EuroG 10k Bead chip and Eurogenomics 75K custom SNP chip), which could make the estimation of ${\varvec{\Gamma}}$ even less accurate. We have also investigated the predictive ability of pedigree BLUP and metafounder pedigree BLUP methods (see Additional file 2: Table S1) and found that these two methods had a higher estimated population accuracy than ssGBLUP, MF-ssGBLUP and BS-ssGBLUP, but also that the estimated genetic variances were much smaller. These are puzzling results, which show that it is necessary to better understand how the estimation of the population accuracy in the LR method performs with imprecisely estimated parameters.

In terms of allele tracing, errors in detecting the breed of origin of alleles can affect a model’s predictive ability especially for a distantly-related crossbred population [15, 30, 48]. In our study, only few such errors were expected since all the alleles on one chromosome should originate from the same breed (either the sire breed or the dam breed) [30]. We also tested the accuracy of allele tracing in a simulated two-way crossbred population, and this was equal to 100% (results not shown). However, in more complicated situations (three-way, four-way, and rotational crossbred populations), our method is not suitable, and a more advanced method for tracing the breed origin of alleles is needed [30, 49].

Overall, MF-ssGBLUP and BS-ssGBLUP had a better predictive ability than ssGBLUP, when the estimated variance components were consistent across the methods. However, more research with larger datasets is needed for investigating the differences between these methods.

## Conclusions

Our results reveal that, for FCR, there are little differences in the estimated genetic parameters of a bivariate model among the ssGBLUP, MF-ssGBLUP, and BS-ssGBLUP methods. However, for ADG, the estimated genetic parameters obtained with BS-ssGBLUP showed a large deviation compared to those with ssGBLUP and MF-ssGBLUP. The values of four estimators implemented in the LR method showed that, for the genetic evaluation for crossbred performance in a two-way crossbred cattle production system, MF-ssGBLUP and BS-ssGBLUP had a better predictive ability than ssGBLUP, when the estimated variance components were consistent across the three methods. In general, compared with BS-ssGBLUP, MF-ssGBLUP is more robust in its superiority over ssGBLUP.

## Supplementary Information

**Additional file 1: Figure S1.** Details of phenotype editing.

**Additional file 2: Table S1.** Bias ($\widehat{\Delta }$), dispersion ($\widehat{\mathrm{b}}$), population accuracy ($\widehat{\mathrm{acc}}$), ratio of population accuracies ($\widehat{\uprho }$) of EBV, average inbreeding coefficient (F) and average relationship (2f) for focal individuals (cut off date, May 1, 2021) and the estimated additive genetic variance in partial dataset(${\upsigma }_{\mathrm{u},\infty }^{2}$) and whole dataset(${\upsigma }_{\mathrm{u}}^{2}$) with different method.

## Data Availability

The phenotypic data is owned by partners of the FutureBeefCross project. The pedigree and genomic data are property of Nordic Cattle Genetic Evaluation Ltd (NAV, Aarhus, Denmark) and Viking Genetics (Randers, Denmark). None of this data is for public distribution.
