# Supplementary material for: Genomic evaluation for two-way crossbred performance in cattle
Source: Genet Sel Evol. 2023 Mar 17;55:17. doi: 10.1186/s12711-023-00792-4 (PMC10022181; doi:10.1186/s12711-023-00792-4)

**Details of phenotype editing**

Raw feed intake data and body weight data from 7 herds have been pre-filtered by the experienced staff in SEGES. Since the feed type was different across 7 herds, we use the dry matter intake instead of the original feed-intake. In the pre-filtered data, daily feed intake and body weight of 4089 two-way crossbred calves have been recorded around one month with an average age of 208 days (standard deviation equals to 34 days) in the beginning of test, and 245 days (standard deviation equals to 33 days) in the end of test. Concerned about that there were gaps between weight date and feed intake date (start weight date and start feed intake date, final weight date and final feed intake date), 10 days gap for each calve were allowed for guaranteeing the measurement accuracy of ADG and FCR while without losing too many information. In terms of the recording of daily feed intake, 5 days gap were allowed for two adjacent feed intake date.

During this period, 1497 calves were removed due to either only with single daily feed intake record or only with single weight record. ADG were calculated based on as the increase in body weight divided by number of days. FCR were calculated as average feed intake divided by average daily gain. After data editing, 2592 crossbred calves with ADG and 2306 crossbred calves with FCR were retained. The distribution of ADG and FCR after editing are shown in below:


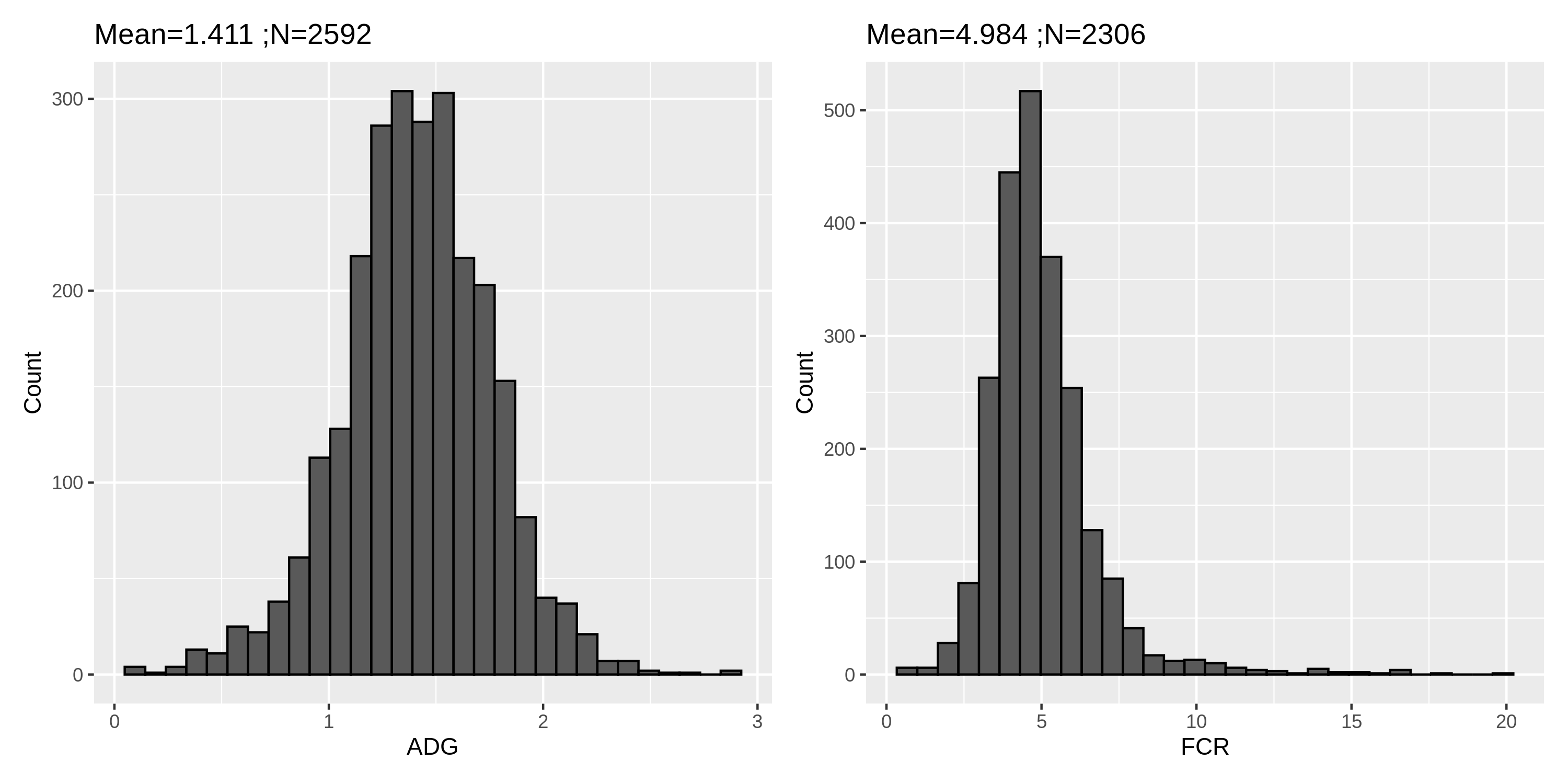

Supplement: Supplementary file 1 — Additional file 1: Figure S1. Details of phenotype editing. [file 12711_2023_792_MOESM1_ESM.docx]
