# Supplementary material for: Genomic evaluation for two-way crossbred performance in cattle
Source: Genet Sel Evol. 2023 Mar 17;55:17. doi: 10.1186/s12711-023-00792-4 (PMC10022181; doi:10.1186/s12711-023-00792-4)
Supplement: Supplementary file 2 — Additional file 2: Table S1. Bias ($\widehat{\Delta }$Δ^), dispersion ($\widehat{\mathrm{b}}$b^), population accuracy ($\widehat{\mathrm{acc}}$acc^), ratio of population accuracies ($\widehat{\uprho }$ρ^) of EBV, average inbreeding coefficient (F) and average relationship (2f) for focal individuals (cut off date, May 1, 2021) and the estimated additive genetic variance in partial dataset(${\upsigma }_{\mathrm{u},\infty }^{2}$σu,∞2) and whole dataset(${\upsigma }_{\mathrm{u}}^{2}$σu2) with different method. [file 12711_2023_792_MOESM2_ESM.docx]

#### Additional file 2 Table S1

#### Bias ($\hat{\boldsymbol{\Delta}}$), dispersion ($\hat{\mathbf{b}}$), population accuracy ($\hat{\mathbf{acc}}$), ratio of population accuracies ($\hat{\boldsymbol{\rho}}$) of EBV, average inbreeding coefficient (F) and average relationship (2f) for focal individuals (cut off date, May 1,2021) and the estimated additive genetic variance in partial dataset($\boldsymbol{\sigma}_{\mathbf{u}\boldsymbol{,\infty}}^{\mathbf{2}}$) and whole dataset($\boldsymbol{\sigma}_{\mathbf{u}}^{\mathbf{2}}$) with different methods

| **Trait** | **Method** | $\hat{\boldsymbol{\Delta}}$ | $\hat{\mathbf{b}}$ | $\hat{\mathbf{acc}}$ | $\hat{\boldsymbol{\rho}}$ | **F** | **2f** | $\boldsymbol{\sigma}_{\mathbf{u}\boldsymbol{,\infty}}^{\mathbf{2}}$ | $\boldsymbol{\sigma}_{\mathbf{u}}^{\mathbf{2}}$ |
| --- | --- | --- | --- | --- | --- | --- | --- | --- | --- |
| ADG | PBLUP | 0.003 | 1.046 | 0.246 | 0.730 | 0 | 0.055 | 0.006 | 0.003 |
|  | MF-PBLUP | 0.003 | 1.041 | 0.287 | 0.727 | 0.797 | 0.929 | 0.005 | 0.002 |
|  | ssGBLUP | 0.009 | 1.101 | 0.232 | 0.633 | 0 | 0.070 | 0.010 | 0.008 |
|  | MF-ssGBLUP | 0.011 | 1.112 | 0.239 | 0.650 | 0.789 | 0.919 | 0.010 | 0.007 |
|  | BS-ssGBLUP | 0.003 | 1.075 | 0.224 | 0.496 | -0.007 | 0.023 | 0.015 | 0.014 |
| FCR | PBLUP | -0.014 | 0.753 | 0.228 | 0.696 | 0 | 0.055 | 0.150 | 0.137 |
|  | MF-PBLUP | -0.014 | 0.749 | 0.267 | 0.695 | 0.797 | 0.929 | 0.111 | 0.107 |
|  | ssGBLUP | -0.040 | 0.707 | 0.208 | 0.630 | 0 | 0.070 | 0.231 | 0.189 |
|  | MF-ssGBLUP | -0.007 | 0.711 | 0.212 | 0.641 | 0.789 | 0.919 | 0.232 | 0.192 |
|  | BS-ssGBLUP | -0.004 | 0.775 | 0.255 | 0.703 | -0.007 | 0.023 | 0.216 | 0.167 |

PBLUP: Pedigree BLUP;

MF-PBLUP: Metafounder PBLUP;

MF-ssGBLUP: Metafounder ssGBLUP;

BS-ssGBLUP: Breed-specific ssGBLUP;

ADG: average daily gain; FCR: feed conversion ratio;
